# The radical scavenging activity of muriolide in physiological environments: mechanistic and kinetic insights into double processes†

**DOI:** 10.1039/d1ra06632c

**Published:** 2021-10-11

**Authors:** Nguyen Thi Hoa, Le Thi Ngoc Van, Quan V. Vo

**Affiliations:** The University of Danang – University of Technology and Education Danang 550000 Vietnam vvquan@ute.udn.vn; Duy Tan University Danang 550000 Vietnam

## Abstract

Muriolide (MO) is a natural lactone that was isolated from *Ranunculus muricatus*. This compound exhibited good antioxidant activity in some experiments; however, the radical scavenging activity of MO in physiological environments has not been studied yet. In this study, the reaction between hydroperoxyl radical and MO was investigated in physiological environments by using density functional theory (DFT) calculations. It was found that MO exhibits excellent antiradical activity in water at physiological pH (*k* = 1.05 × 10^8^ M^−1^ s^−1^) by the single electron transfer mechanism of the anion state. However, the activity in lipid media is moderate with *k* = 2.54 × 10^4^ M^−1^ s^−1^ and is defined by the formal hydrogen transfer pathway. The antiradical reactions can occur in double processes; however, the first reaction may define the HOO˙ radical scavenging activity of MO. Compared with typical natural antioxidants, the antiradical activity of MO against HOO˙ radicals is slightly lower than Trolox in pentyl ethanoate. However, the activity of MO is approximately 808 times faster than that of the reference in aqueous solution. Thus, the data suggest that MO is a promising natural radical scavenger in the physiological environment.

## Abstract 2

Muriolide, which is a natural lactone that was isolated from *Ranunculus muricatus*, is a promising natural radical scavenger in the physiological environment.

## Graphical abstract

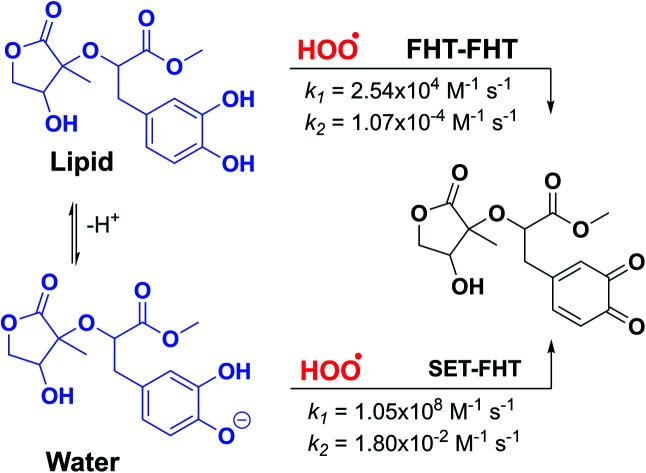

## 1. Introduction

*Ranunculus muricatus*, which belongs to the genus *Ranunculus* (Ranunculaceae), is known as spiny fruit buttercup in Asia, Australia, South America, and Europe.^1^ The plant has been used as a traditional drug to treat urinary infections, jaundice, diarrhea, dysentery, eczema, leprosy, and ringworm infection.^2–4^*R. muricatus* is also used as a remedy for coughs and asthma and a deworming agent for all types of livestock.^5,6^ Studies showed that *R. muricatus* exhibited cytotoxic, antibacterial, antifungal, and particularly antioxidant properties.^7,8^ The antioxidant activity of *R. muricatus* may be related to phenolic compounds such as flavonoids, flavonoid glycosides, and lactones found in the plant.^7,9,10^

**Fig. 1:**
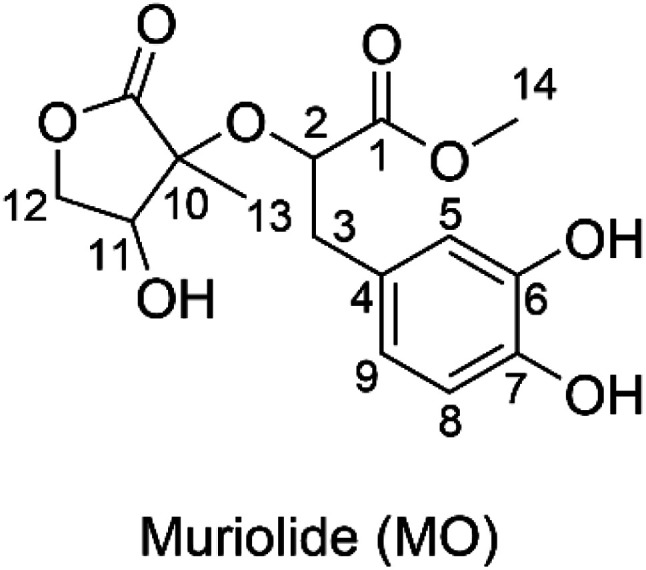
Molecular structure of muriolide (MO).

Muriolide (MO, Fig. 1) is a natural lactone that has been isolated from *Ranunculus muricatus*.^9^ This compound exhibited good antioxidant activity against the DPPH radical scavenging activity (IC_50_ = 56.9 μM) and lipoxygenase enzyme testing (IC_50_ = 68.3 μM). Thus the radical scavenging activity of MO, particularly in the physiological systems, needs to be investigated; however, this issue has not been mentioned yet. Previous studies have shown that the computational method is one of the most convenient means of examining the relationship between structures and biological activity in the development of new medicines, such as antioxidants with increased activities.^11–15^ Therefore, in this study, the antiradical activity of MO was thoroughly evaluated in double processes by using quantum calculations. In addition, the effects of solvents and molecule structure on the activity were also considered.

## 2. Computational details

Calculations were carried out with the Gaussian 09 suite of programs^16^ by using the M06-2X/6-311++G(d,p) method.^17^ The M06-2X functional is one of the most reliable methods to study thermodynamics and the kinetics of radical reactions,^18–20^ and widely used to evaluate the antiradical scavenging activity in solvents (water for polar media and pentyl ethanoate for lipid environments)^15,21^ with low errors compared to experimental data (*k*_calc_/*k*_exp_ ratio = 1–2.9).^21–25^ The kinetic calculations were performed following the quantum mechanics-based test for the overall free radical scavenging activity (QM-ORSA) protocol,^12,21^ with the SMD method for solvent effects,^26^ by the Eyringpy code.^24,27^ All of the species have been optimized directly in the specific environments, *i.e.* gas phase, pentyl ethanoate and water. The rate constant was calculated by using the conventional transition state theory (TST) and 1 M standard state at 298.15 K.^24,25,27–32^1
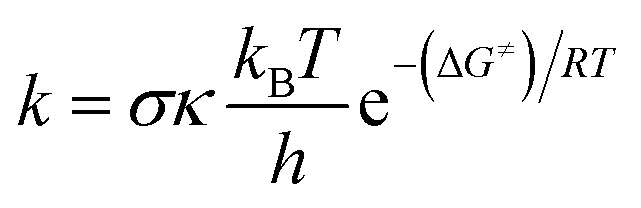
where *σ* is the reaction symmetry number,^33,34^*κ* contains the tunneling corrections calculated using the Eckart barrier,^35^*k*_B_ is the Boltzmann constant, *h* is the Planck constant, Δ*G*^≠^ is the Gibbs free energy of activation. The Marcus Theory was used to estimate the reaction barriers of single electron transfer (SET) reactions.^36–38^ The free energy of reaction Δ*G*^≠^ for the SET pathway was computed following the eqn (2) and (3).2
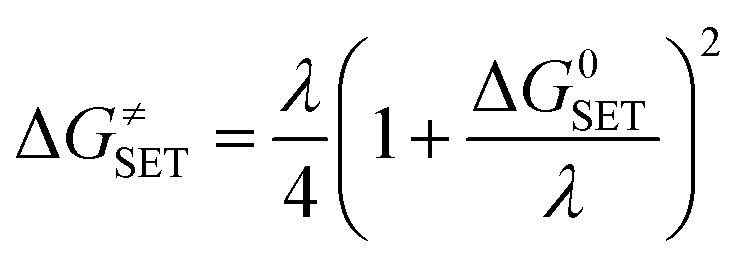

3 *λ* ≈ Δ*E*_SET_ − Δ*G*^0^_SET_

where Δ*G*_SET_ is the Gibbs energy of reaction, Δ*E*_SET_ is the non-adiabatic energy difference between reactants and vertical products for SET.^39,40^

For rate constants that were close to the diffusion limit a correction was applied to yield realistic results.^21^ The apparent rate constants (*k*_app_) were calculated following the Collins–Kimball theory in the solvents at 298.15 K;^41^ the steady-state Smoluchowski rate constant (*k*_D_) for an irreversible bimolecular diffusion-controlled reaction was calculated following the literature as corroding to eqn (4) and (5).^21,42^4
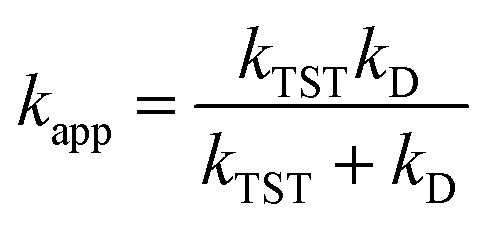

5 *k*_D_ = 4π*R*_AB_*D*_AB_*N*_A_

where *R*_AB_ is the reaction distance, *N*_A_ is the Avogadro constant, and *D*_AB_ = *D*_A_ + *D*_B_ (*D*_AB_ is the mutual diffusion coefficient of the reactants A and B),^41,43^ where *D*_A_ or *D*_B_ is estimated using the Stokes–Einstein formulation (6).^44,45^6
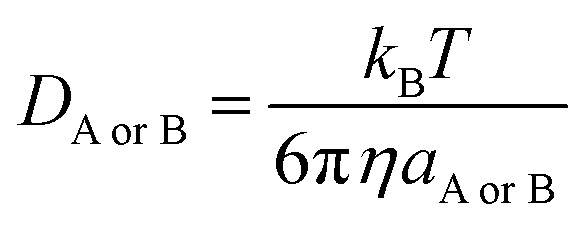
*η* is the viscosity of the solvents (*i.e. η*(H_2_O) = 8.91 × 10^−4^ Pa s, *η*(pentyl ethanoate) = 8.62 × 10^−4^ Pa s) and *a* is the radius of the solute that was obtained in Gaussian calculations.

The solvent cage effects were included following the corrections proposed by Okuno,^46^ adjusted with the free volume theory according to the Benson correction^21,47–49^ to reduce over-penalizing entropy losses in solution. All transition states were characterized by the existence of only one single imaginary frequency. Intrinsic coordinate calculations (IRCs) were performed to ensure that each transition state (TS) is connected correctly with the pre-complex (RC) and post-complex (PC).

## 3. Results and discussion

### 3.1 The gas phase evaluation

**Fig. 2:**
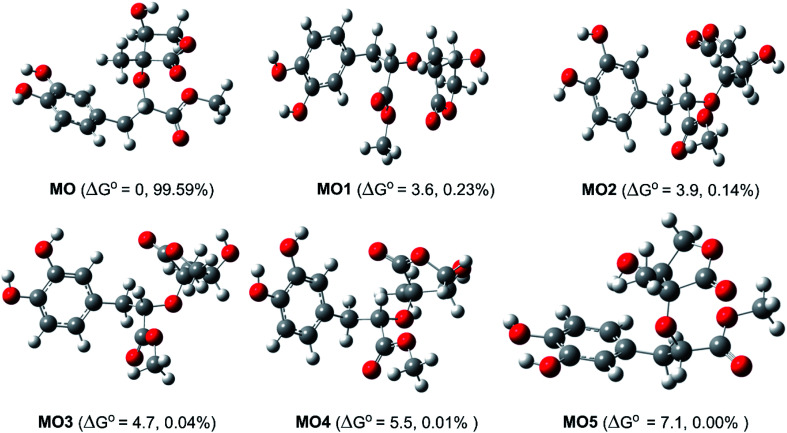
The typical MO conformers and the relative free energy Δ*G*° (compared with MO, kcal mol^−1^).

Study of the structure of the MO showed that the molecule can adopt multiple conformational structures. Thus, as an initial step, the possibility of MO conformers was examined^50^ and then the M06-2X/6-311++G(d,p) method was used to analyze the six lowest electronic energy conformers (Fig. 2). It was found that, the lowest Δ*G*° value was observed at MO, those for MO1–MO5 were higher than that of MO about 3.6–7.1 kcal mol^−1^. With the Maxwell–Boltzmann distribution,^51,52^ it was found that MO is the dominant conformer (99.59%) in the relative tautomer populations, and this conformer has therefore been used in further studies.

The radical scavenging activity of MO was evaluated against HOO˙ radical. This radical species is the simplest of the biologically most important of the ROO˙, *i.e.* peroxy radicals, and effective scavenging of these is sufficient to reduce oxidative stress in biological systems.^53^ The HOO˙ radical, which is moderately reactive and one of the main antioxidant objectives,^54^ has been widely used as a reference radical for modeling antioxidant activity in lipid and polar environments.^12,21,48,55^

To understand how MO scavenges free radicals, the antioxidant reactivity of MO was first evaluated following the three typical antioxidant mechanisms, including formal hydrogen transfer (FHT), sequential proton loss electron transfer (SPLET), and single electron transfer proton transfer (SETPT).^11^ Those are defined by the thermochemical parameters (bond dissociation enthalpy (BDE), proton affinity (PA), and ionization energy (IE), respectively). Previous studied showed that the radical adduct formation (RAF) of HOO˙ radical was not favored for the π system of aromatic rings^48,56–58^ such as in MO and therefore this mechanism was not considered in this study. Therefore, the thermodynamic parameters in the gas phase of MO were computed and results are presented in Table 1.

**Table 1:** Calculated thermodynamic parameters (kcal mol^−1^) of the MO + HOO˙ *via* FHT, SP and SET reactions

Positions	FHT		SP		SET	
	BDE	Δ*G*°	PA	Δ*G*°	IE	Δ*G*°
C2–H	88.4	3.6			185.0	162.1
C3–H	93.0	7.6				
O6–H	79.6	−5.4	334.6	183.5		
O7–H	79.0	−6.0	332.2	181.1		
O11–H	104.0	18.4	353.8	201.9		
C11–H	98.0	11.4				
C12–H	98.1	11.8				

The lowest BDE values are presented at the phenolic groups, including O6(O7)–H bonds with BDE = 79.6 and 79.0 kcal mol^−1^, respectively. The BDE of the alcohol group (O11–H) is highest at 104.0 kcal mol^−1^, and this is about ∼6 and 15.6 kcal mol^−1^ higher than those of the C11(12)–H bonds and C2–H bond, respectively. Calculated thermodynamic parameters in studied solvents (Table S1, ESI†) indicated that the lowest BDE values were also obtained at the O6(7)–H bonds (BDE(O6(7)–H) = 79.7, 79.0 and 82.6, 82.0 in pentyl ethanoate and water, respectively). Thus the results suggest that the O6(O7)–H bonds will define the H-abstraction of MO following the FHT mechanism. It is clear from Table 1 that the PA and IE values are much higher than the BDEs. The lowest PA (PA(O7–H) = 332.2 kcal mol^−1^) and IE values are around 4.21 and 2.34 times greater than the lowest BDE. Thus the radical scavenging of MO in the gas phase may be followed the FHT pathway rather than the SETPT and SPLET mechanisms. This result was confirmed by investigating the Gibbs free energies of the reaction between MO and HOO˙ radicals (Table 1).^25,59^ The HOO˙ trapping activity of MO is spontaneous for FHT at O6(7)–H bonds (Δ*G*° = −5.4 and −6.0 kcal mol^−1^, respectively), whereas the other reactions are unspontaneous with high positive Δ*G*° values. Based on the calculated data, the MO + HOO˙ reaction may only follow the FHT mechanism, and thus this pathway should be investigated in the kinetic study.

**Fig. 3:**
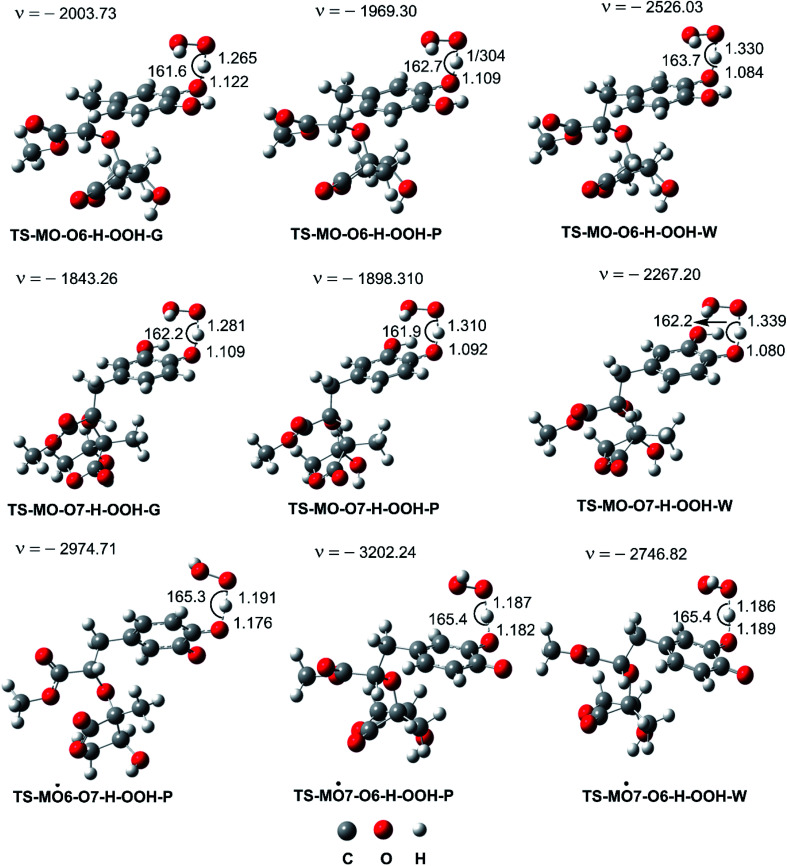
Optimized geometries of FHT TSs of the HOO˙ + MO reaction (G: gas phase, W: water, P: pentyl ethanoate).

In the next step evaluation of the HOO˙ trapping activity of MO, the kinetics of the HOO˙ + MO reaction following the primary mechanism (FHT at O6(7)–H bonds) in the gas phase were computed according to the (QM-ORSA) protocol,^12,21^ the results are shown in Table 2 and Fig. 3. It was found that MO exhibited moderate hydroperoxyl antiradical activity with *k*_Eck_ = 1.63 × 10^6^ and 2.71 × 10^6^ M^−1^ s^−1^ for the O6–H and O7–H bonds, respectively. These reactions contribute about 37.5 and 62.5% in the overall rate constant (*k*_overall_ = 4.34 × 10^6^ M^−1^ s^−1^). However, the *k*_overall_ of the HOO˙ + MO reaction in the gas phase is ∼4.3 times lower than that of Trolox (*k* = 1.87 × 10^7^ M^−1^ s^−1^).^60^ Hence, it appears to suggest that the hydroperoxyl antiradical activity of MO in nonpolar media might be lower than that of Trolox.

**Table 2:** Calculated Δ*G*^≠^ (kcal mol^−1^), tunneling corrections (*κ*), *k*_Eck_ (M^−1^ s^−1^) and branching ratios (*Γ*, %) for the HOO˙ + MO reaction

Positions	Δ*G*^≠^	*κ*	*k*_Eck_	*Γ*
O6–H	12.0	169.0	1.63 × 10^6^	37.5
O7–H	11.3	77.7	2.71 × 10^6^	62.5
***k***_**overall**_			**4.34 × 10** ^**6**^	

### 3.2 The HOO˙ radical trapping activity of MO in physiological environments

#### 3.2.1 The first process

**Fig. 4:**
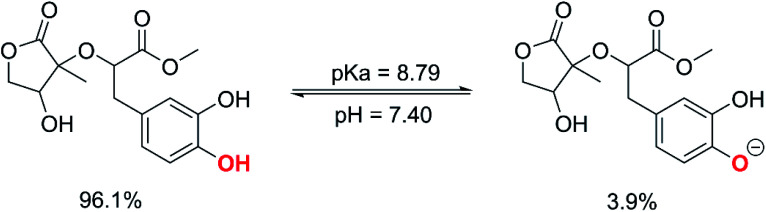
The deprotonation of MO at pH = 7.4.

In aqueous environments, the radical scavenging activity of acidic species is typically dominated by the activity of the ionic forms.^18,59^ Therefore, the protonation state of MO was first evaluated at physiological pH to find the most likely radical scavenging reactions. The thermodynamic section and the calculations for water medium (Table S1, ESI,† (PA(O6–H) = 43.1 kcal mol^−1^), PA(O7–H) = 42.9 kcal mol^−1^) showed that deprotonation was the easiest at the O7–H bond; thus, the p*K*_a_ value of MO was computed for the O7–H bond based on the literature^59,61^ and shown in Fig. 4.

**Fig. 5:**
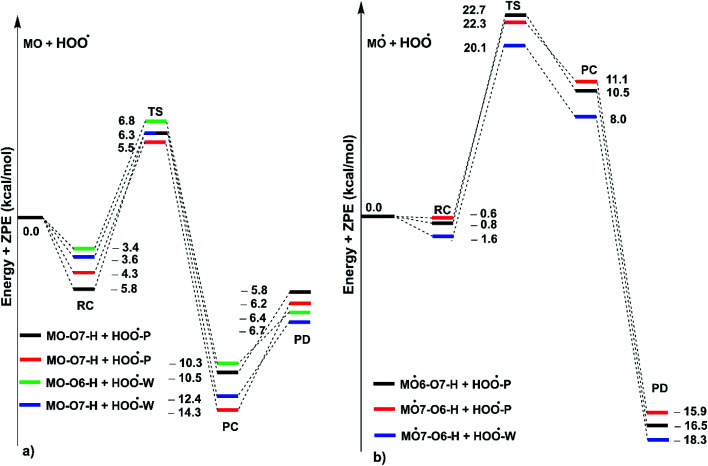
PES of reactions between MO and HOO˙ in pentyl ethanoate (P) and water (W) in double processes ((a): the first step; (b) the second step; RC: pre-complexes, TS: transition states, PC: post-complexes, PD: products).

The calculated p*K*_a_ value was p*K*_a_ = 8.79. Therefore, in pH = 7.40 aqueous solutions, MO exists in two states, including the neutral (HA, 96.1%) and anion (A^−^, 3.9%) states. Hence, these states were used in the kinetic study of the HOO˙ trapping activity of MO in water at pH = 7.4. The overall rate constant (*k*_overall_) of HOO˙ + MO reaction in the first antiradical process were calculated according to eqn (7) and (8); the results are presented in Table 3 and Fig. 3, while the potential energy surfaces of the HOO˙ + MO reaction following the FHT pathway is shown in Fig. 5.

**Table 3:** Calculated Δ*G*^≠^ (kcal mol^−1^) and rate constants (*k*_app_, *k*_f_, and *k*_overall_ M^−1^ s^−1^) at 298.15 K, in the first process of MO + HOO˙ reaction

Mechanisms		Pentyl ethanoate				Water					
		Δ*G*^≠^	*κ*	*k*_app_	*Γ*	Δ*G*^≠^	*κ*	*k*_app_	*f*	*k*_f_	*Γ*
SET	MO–O7^−^					4.3	16.3^a^	2.70 × 10^9^	0.039	1.05 × 10^8^	100.0
FHT	O6–H	15.3	175.3	6.40 × 10^3^	25.2	16.1	932.1	8.50 × 10^3^	0.961	8.17 × 10^3^	0.0
	O7–H	14.2	79.5	1.90 × 10^4^	74.8	15.0	312.8	2.10 × 10^4^	0.961	2.02 × 10^4^	0.0
***k*_overall_**				**2.54 × 10^4^**						**1.05 × 10^8^**	

a The nuclear reorganization energy (*λ*, kcal mol^−1^); *f* = %A^−^/100; *k*_f_ = *fk*_app_; *Γ* = *k*_f_ × 100/*k*_overall_.

In lipid medium:

7 *k*_overall_ = *k*_app_(FHT(O6–H)-neutral) + *k*_app_(FHT(O7–H)-neutral)

In the aqueous medium:

8 *k*_overall_ = *k*_f_(SET-anion) + *k*_f_(FHT(O6–H)-neutral) + *k*_f_(FHT(O7–H)-neutral)

As shown in Fig. 5, in the first antiradical process, the reaction proceeds *via* the RCs that are more stable in terms of energy than the reactants about 3.4–5.8 kcal mol^−1^. Then, the reaction can proceed to TSs from the RCs (the energy barriers around 12.0–17.0 kcal mol^−1^) before bottoming the lowest energy points (PCs) and forming the products. The energy barriers for the MO–O6–H + HOO˙ reaction in water and pentyl ethanoate are higher (from 0.3 to 2.1 kcal mol^−1^, respectively) than those of the MO–O7–H + HOO˙ reaction. This suggests that the H-abstraction of the O7–H bond against HOO˙ radicals should be faster than that of the O6–H bond.

It is clear from the Table 3 that the hydroperoxyl radical trapping activity of MO in pentyl ethanoate is moderate with the *k*_overall_ = 2.54 × 10^4^ M^−1^ s^−1^ by the H-abstractions of the O6–H (*Γ* = 25.2%) and O7–H bonds (*Γ* = 74.8%). In contrast, MO exhibits an excellent HOO˙ trapping activity in the polar medium with the *k*_overall_ = 1.05 × 10^8^ M^−1^ s^−1^. This process was defined by the SET reaction of the anion state (MO–O7^−^, *Γ* ∼ 100%). The rate constants for the FHT reaction of the O6(7)–H bonds against HOO˙ radical in water are *k*_f_ = 8.17 × 10^3^ (2.02 × 10^4^) M^−1^ s^−1^, whereas these reactions make negligible contributions (∼0%) to the overall HOO˙ antiradical activity of MO. However, the reaction at the O7–H bond is faster than that of the O6–H bond in all of the studied media. This is in good agreement with the PES analysis results. Compared with the reference antioxidant Trolox (*k* = 1.00 × 10^5^ and 1.30 × 10^5^ M^−1^ s^−1^ in pentyl ethanoate and water, respectively),^60^ the HOO˙ trapping activity of MO is fairly lower in the lipid medium but about 808 times higher in water at physiological pH.

#### 3.2.2 The second process of the radical scavenging

To gain further insights into the antioxidant of MO in the physiological environments, the hydroperoxyl radical scavenging activity of MO intermediates (the second antiradical process of MO) in pentyl ethanoate and water was investigated. As shown in the first reaction step, the primary intermediates of MO + HOO˙ reaction in pentyl ethanoate were MO–O6˙ (25.2%) and MO–O7˙ (74.8%) radicals, while that for the aqueous solution was MO–O7˙ (100.0%). Thus these intermediates were used as reactants for the second reaction against HOO˙ radicals. The thermodynamic parameters (BDEs, IE) were computed for the most active positions and are shown in Table 4.

**Table 4:** Calculated thermodynamic parameters (kcal mol^−1^) of the intermediates + HOO˙ *via* FHT and SET mechanisms in pentyl ethanoate (P) and water (W)

Solvents	Intermediates	Positions	FHT		SET	
			BDE	Δ*G*°	IE	Δ*G*°
P	MO–O6˙	O7–H	69.2	−16.4	145.9	75.8
	MO–O7˙	O6–H	69.9	−16.0	143.9	72.5
W	MO–O7˙	O6–H	71.1	−18.8	132.0	26.3

As shown in Table 4, the BDE values of the most active positions (O6–H and O7–H bonds) are in the range of 69.2 to 71.1 kcal mol^−1^, much lower than those of MO in the first step (Table 1). At the same time, the calculated IE values of intermediates (MO–O6˙, MO–O7˙) are 132.0–145.9 kcal mol^−1^; however, the SET mechanism is not favored for the intermediates due to the large positive Δ*G*° (Δ*G*° = 26.3–75.8 kcal mol^−1^). Thus the HOO˙ radical scavenging activity of intermediates was defined by the FHT pathway (Δ*G*° < 0, Table 4); thus, these reactions were used for kinetic investigating.

Previous studies also showed that the reaction between antiradical intermediates with radical *i.e.*, HO˙ and HOO˙ most probably proceeds through triplet transition states,^55,62^ and thus, the result was used to evaluate the mechanism and kinetics of the second antiradical reaction of MO. The potential energy surfaces are shown in Fig. 5, whereas the possible mechanisms and kinetic data are presented in Fig. 5 and Table 4, respectively.

**Fig. 6:**
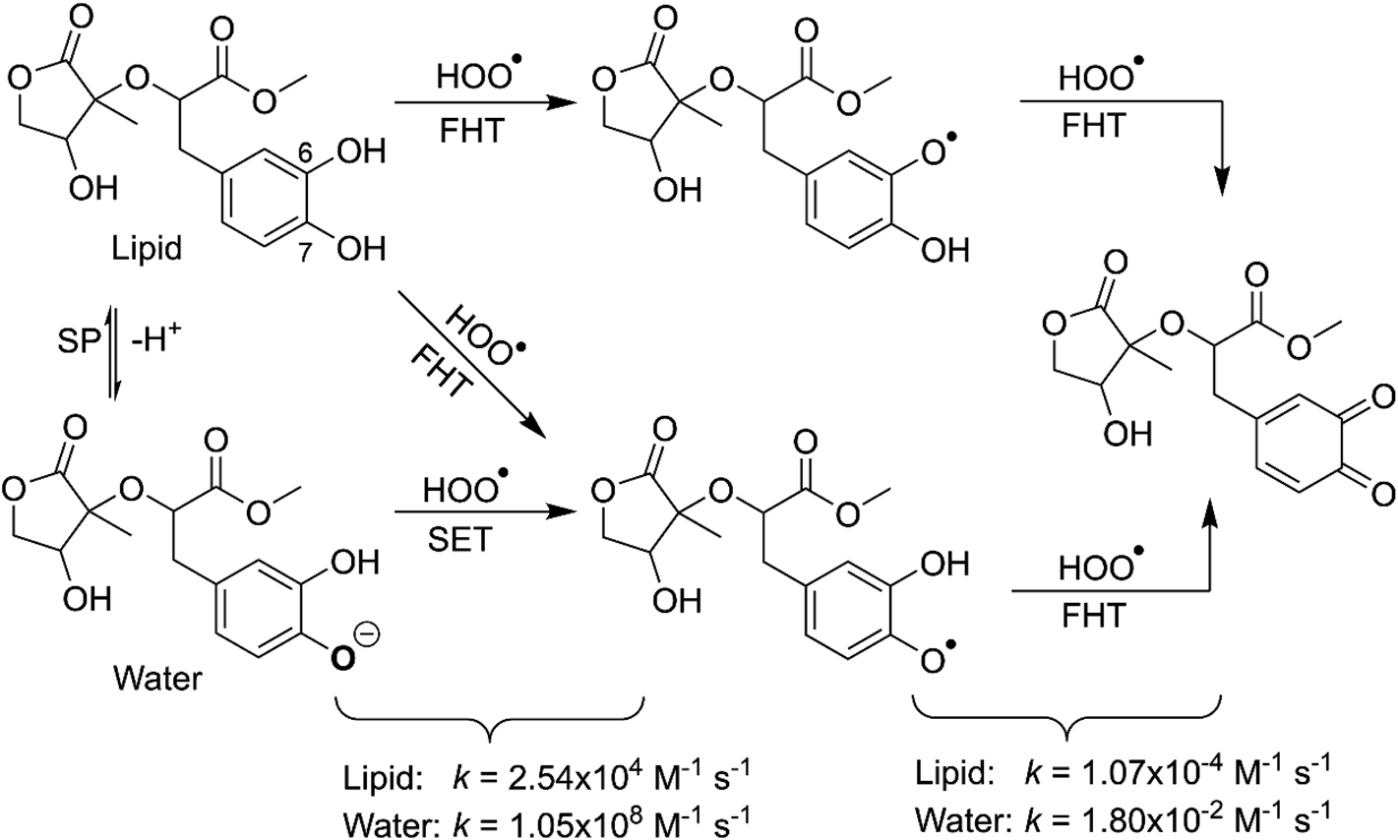
The possible mechanisms for the HOO˙ + MO reaction in the physiological environment.

As shown in Fig. 5, the reactions of intermediates (MO–O6˙ and MO–O7˙) and HOO˙ radicals proceed *via* RCs, TSs and PCs, while the PC species are less stable in terms of energy than the reactants. This is in line with previous studies in phenolic compounds.^55,62^ The energy barriers of the reactions are about 20.1–22.7 kcal mol^−1^, which is much higher than those of the first step (the energy barriers around 12.0–17.0 kcal mol^−1^, Table 5). The overall rate constants of the intermediates (MO–O6˙ or MO–O7˙) + HOO˙ reactions in pentyl ethanoate is 1.07 × 10^−4^ M^−1^ s^−1^, while that for the aqueous solution is 1.80 × 10^−2^ M^−1^ s^−1^. Thus the HOO˙ radical scavenging activity in the second reaction step of MO following the FHT pathway is about 10^6^–10^8^ times lower than those of the first reaction step (Fig. 6), despite of the fact that the BDE(O–H) values at the intermediates (BDEs = 69.2–71.1 kcal mol^−1^, Table 4) are lower than those of MO (Table 1) by about 10 kcal mol^−1^, and the Gibbs free energies for the intermediates (MO–O6˙ or MO–O7˙) + HOO˙ reactions are Δ*G*° = −16.0 to −18.8 kcal mol^−1^ (Table 4). These results suggest that the HOO˙ radical scavenging of MO at the second process is supported by the thermodynamic properties (the low BDE values and Δ*G*° < 0); however, this reaction hardly occurs due to the low rate constant values. Thus the antiradical activity should be considered in both thermodynamic and kinetic data rather than based on thermodynamic considerations alone. Based on the calculated data, the HOO˙ trapping activity of MO in nonpolar and polar environments was mainly defined by the first step.

**Table 5:** Calculated Δ*G*^≠^ (kcal mol^−1^) and rate constants (*k*_f_, and *k*_overall_ M^−1^ s^−1^) at 298.15 K, in the second process of MO + HOO˙ reaction^a^

Mechanisms	Reactions	Pentyl ethanoate		Water	
		Δ*G*^≠^	*k*_f_	Δ*G*^≠^ (*λ*)	*k*_f_
SET	MO7˙ + HOO˙			27.3 (17.7)	5.60 × 10^−8^
FHT	MO7˙–O6–H + HOO˙	30.5	1.06 × 10^−4^	29.1	1.80 × 10^−2^
	MO6˙–O7–H + HOO˙	30.9	1.20 × 10^−6^		
***k***_**overall**_			**1.07 × 10** ^**−4**^		**1.80 × 10** ^**−2**^

a *k*_f_ = *fk*_app_; in water *f*(intermediate) = 1.00; in pentyl ethanoate *f*(MO–O7˙) = 0.748, *f*(MO–O6˙) = 0.252.

## 4. Conclusion

The hydroperoxyl radical scavenging activity of muriolide in the physiological environment has been successfully investigated *in silico*. The result showed that MO exhibited moderate activity (*k* = 2.54 × 10^4^ M^−1^ s^−1^) in the nonpolar media, whereas the activity was excellent with *k* = 1.05 × 10^8^ M^−1^ s^−1^ in water under the physiological pH. The antiradical reactions could occur in two steps; however, the first step reaction defined the HOO˙ radical scavenging activity of MO. In nonpolar conditions, the FHT mechanism *via* the O6–H and O7–H bonds determined the antiradical activity, whereas the SET mechanism of the anionic state defined the activity in the polar medium. The HOO˙ + MO reaction in pentyl ethanoate is slightly lower than Trolox, but it is approximately 808 times faster than that of the reference in the aqueous solution. Thus, MO is an effective radical scavenger in the physiological environment.

## Conflicts of interest

There are no conflicts to declare.

## Supplementary Material

RA-011-D1RA06632C-s001
