# Supplementary material for: The radical scavenging activity of muriolide in physiological environments: mechanistic and kinetic insights into double processes
Source: RSC Adv. 2021 Oct 11;11(53):33245–52. doi: 10.1039/d1ra06632c (PMC9042307; doi:10.1039/d1ra06632c)
Supplement: RA-011-D1RA06632C-s001 [file RA-011-D1RA06632C-s001.pdf]

Electronic Supplementary Material (ESI) for RSC Advances.
This journal is © The Royal Society of Chemistry 2021

Supporting Information (SI)

The Radical Scavenging Activity of Muriolide in Physiological Environments: Mechanistic and kinetic insights into Double Processes

Nguyen Thi Hoa[1] Le Thi Ngoc Van[2] and Quan V. Vo[1*]

[1]The University of Danang - University of Technology and Education, Danang 550000, Vietnam.

[2]Duy Tan University, Danang 550000, Vietnam

**Corresponding authors: vvquan@ute.udn.vn;*

Table of Contents

Table S1. Calculated thermodynamic parameters (kcal/mol) of the MO in the studied solvents S2

Table S2: The Cartesian coordinates and energies of TS of the reaction between MO with HOO in the studied environments at the M06-2X/6-311++G(d,p) level of theory. S3

Table S1. **Calculated thermodynamic parameters (kcal/mol) of the MO in the studied solvents**

**Positions**	**BDE**		**PA**		**IE**	
	**P**	**W**	**P**	**W**	**P**	**W**
C2–H	89.5	91.0			136.6	109.2
C3–H	91.7	92.0				
O6–H	79.7	82.6	78.6	43.1		
O7–H	79.0	82.0	78.4	42.9		
O11–H	104.3	132.7	97.1	53.6		
C11–H	97.3	98.2				
C12–H	99.0	101.5				
P: pentyl ethanoate; W: water						

Table S2: The Cartesian coordinates and energies of TS of the reaction between MO with HOO in the studied environments at the M06-2X/6-311++G(d,p) level of theory.

**Name**	**TS-MO-O6-H-OOH (Gas phase)**
Cartesian Coordinates	Frequency and Energy
O 3.90414700 1.39711900 0.10297500<br>C 3.33927500 1.83320300 -1.15058200<br>C 1.91381100 2.24390600 -0.79342000<br>C 1.55228700 1.16032700 0.23418400<br>C 2.92878100 0.89743500 0.88840200<br>O 1.19081200 0.03080400 -0.55836000<br>C 1.14433900 -1.22605800 0.08008000<br>C 2.40856100 -2.04827200 -0.15892300<br>C -0.09571200 -1.97495500 -0.41721600<br>C -1.34151600 -1.24975400 0.01611100<br>O 3.38577600 -1.33545300 -0.71882700<br>C 4.65498400 -1.99267000 -0.80966500<br>O 2.50643500 -3.20109600 0.16032700<br>O 3.13740400 0.34503500 1.92611100<br>O 1.85800400 3.54700700 -0.26046900<br>C -1.81923800 -1.40636600 1.33336500<br>C -2.90577200 -0.69095100 1.80664700<br>C -3.54034200 0.21004500 0.95714900<br>C -3.08578400 0.38134700 -0.37868100<br>C -1.98474700 -0.36779800 -0.82888500<br>O -3.72236900 1.25448400 -1.13640800<br>O -4.62211000 0.89410700 1.37243300<br>C 0.48054900 1.52856700 1.23676100<br>H 3.35607600 0.99402600 -1.84701700<br>H 3.93916100 2.66398200 -1.51656700<br>H 1.23166200 2.21110700 -1.64143200<br>H 1.08251200 -1.11402500 1.16899100<br>H -0.04813600 -2.03998200 -1.50680600<br>H -0.06571100 -2.98891500 -0.01390600<br>H 5.33226700 -1.26160900 -1.24276900<br>H 4.99043800 -2.28389300 0.18544300<br>H 4.57592400 -2.87639800 -1.44222600<br>H 2.57616000 3.67021600 0.36949300<br>H -1.31757300 -2.10610100 1.99474900<br>H -3.27248000 -0.81265800 2.81816400<br>H -1.64675400 -0.21246700 -1.84688200<br>H -4.70256700 0.80708700 -1.45045400<br>H -4.84485100 1.54376000 0.68820900<br>H 0.35545200 0.73773300 1.97816500<br>H 0.74771500 2.44500600 1.76258900<br>H -0.46458300 1.68371400 0.71220700<br>H -5.72701400 -0.98246400 0.04769400<br>O -5.76547000 0.12058200 -1.44647900<br>O -5.37194700 -1.03585700 -0.85500500	Zero-point correction= 0.340015 (Hartree/Particle)<br>Thermal correction to Energy= 0.365063<br>Thermal correction to Enthalpy= 0.366007<br>Thermal correction to Gibbs Free Energy= 0.282143<br>Sum of electronic and zero-point Energies= -1334.665694<br>Sum of electronic and thermal Energies= -1334.640646<br>Sum of electronic and thermal Enthalpies= -1334.639702<br>Sum of electronic and thermal Free Energies= -1334.723566
**Name**	**TS-MO-O6-H-OOH (Pentyl ethanoate)**
Cartesian Coordinates	Frequency and Energy
O 3.98603000 1.28150200 -0.00210100<br>C 3.37701800 1.78017200 -1.21609800<br>C 1.98388100 2.22906700 -0.78789100<br>C 1.63844100 1.16615500 0.26890200	Zero-point correction= 0.339348 (Hartree/Particle)<br>Thermal correction to Energy= 0.364412<br>Thermal correction to Enthalpy= 0.365356<br>Thermal correction to Gibbs Free Energy= 0.282037

C	3.03818300	0.83207400	0.83342300	Sum of electronic and zero-point Energies= -1334.699943
O	1.16556000	0.05498400	-0.48958500	Sum of electronic and thermal Energies= -1334.674879
C	1.10321600	-1.19683300	0.15654500	Sum of electronic and thermal Enthalpies= -1334.673935
C	2.35614400	-2.03781000	-0.07382200	Sum of electronic and thermal Free Energies= -1334.757254
C	-0.14155400	-1.93106200	-0.35385600	
C	-1.38351800	-1.18798100	0.06133300	
O	3.25306600	-1.42804600	-0.84167600	
C	4.51475600	-2.09489000	-0.98568800	
O	2.50224100	-3.13015700	0.40937200	
O	3.28740800	0.26194900	1.85721100	
O	1.97577800	3.54327800	-0.27707900	
C	-1.85685300	-1.30455500	1.38577000	
C	-2.94663000	-0.58192500	1.83880400	
C	-3.59009400	0.28735300	0.96201100	
C	-3.14122500	0.41335900	-0.37954200	
C	-2.03390500	-0.33711900	-0.80988800	
O	-3.79219300	1.25566100	-1.16908400	
O	-4.66626500	0.98605400	1.36328100	
C	0.65456400	1.59318600	1.33550400	
H	3.33740600	0.96465400	-1.93865100	
H	3.99159800	2.59844200	-1.58642000	
H	1.26427000	2.19928500	-1.60451100	
H	1.02636700	-1.08366300	1.24332200	
H	-0.08344700	-2.00561600	-1.44239800	
H	-0.13822400	-2.94081400	0.06163300	
H	5.11632700	-1.45277900	-1.62432100	
H	4.98730200	-2.21001400	-0.00966000	
H	4.37628300	-3.07187300	-1.44864600	
H	2.69606000	3.65419400	0.35553400	
H	-1.34938500	-1.97967100	2.06808800	
H	-3.30708700	-0.67601500	2.85611700	
H	-1.70234200	-0.21892900	-1.83549200	
H	-4.72972500	0.78047300	-1.48711200	
H	-4.89460200	1.62147400	0.66685100	
H	0.49175600	0.79280900	2.05931000	
H	1.02532900	2.46311300	1.87905600	
H	-0.29710500	1.85059200	0.86443500	
H	-5.76298800	-1.03285400	0.00709700	
O	-5.78437700	0.01543400	-1.53413900	
O	-5.36254200	-1.09415300	-0.87836100	
**Name**				**TS-MO-O6-H-OOH (Water)**
Cartesian Coordinates				Frequency and Energy
O	3.97648900	1.28018500	-0.03145200	Zero-point correction= 0.339615 (Hartree/Particle)
C	3.37199000	1.82491600	-1.23338000	Thermal correction to Energy= 0.364436
C	1.95666300	2.21975200	-0.82198300	Thermal correction to Enthalpy= 0.365381
C	1.63130700	1.16578100	0.25294000	Thermal correction to Gibbs Free Energy= 0.283248
C	3.03340000	0.83764000	0.80372600	Sum of electronic and zero-point Energies= -1334.711578
O	1.15824700	0.03727600	-0.48633100	Sum of electronic and thermal Energies= -1334.686756
C	1.09854300	-1.20562200	0.18630000	Sum of electronic and thermal Enthalpies= -1334.685812
C	2.35581900	-2.03578600	-0.03632400	Sum of electronic and thermal Free Energies= -1334.767944
C	-0.14653100	-1.95203500	-0.30356300	
C	-1.37780400	-1.18203400	0.09317600	
O	3.22493400	-1.45771000	-0.84771500	
C	4.49415600	-2.11552600	-1.00591800	

O 2.53069200 -3.10688300 0.49960300<br>O 3.29632200 0.25898300 1.82719300<br>O 1.87331500 3.54926700 -0.35131900<br>C -1.80184400 -1.19167800 1.44013600<br>C -2.87247700 -0.43137400 1.87188900<br>C -3.54999500 0.36954400 0.95434100<br>C -3.14817500 0.38739800 -0.40642200<br>C -2.05829400 -0.39924400 -0.81704500<br>O -3.81604800 1.17521500 -1.25115700<br>O -4.60107400 1.11170300 1.35366200<br>C 0.66033100 1.59778500 1.32806700<br>H 3.37623500 1.04387900 -1.99352900<br>H 3.97206600 2.67534700 -1.54745700<br>H 1.25419000 2.13484600 -1.64917700<br>H 1.02444200 -1.07198900 1.26996500<br>H -0.09038500 -2.06186900 -1.38878800<br>H -0.14860800 -2.94541400 0.14955400<br>H 5.06762800 -1.48160800 -1.67644400<br>H 4.98676000 -2.19514600 -0.03687500<br>H 4.35006400 -3.10393900 -1.44051000<br>H 2.53525700 3.70378700 0.33545500<br>H -1.26675200 -1.80917400 2.15470000<br>H -3.19599100 -0.44236800 2.90552400<br>H -1.76292100 -0.36205300 -1.86003500<br>H -4.75495700 0.69389700 -1.49857300<br>H -4.91203000 1.64686100 0.60605300<br>H 0.51604600 0.80450600 2.06367200<br>H 1.03997200 2.47751800 1.84929000<br>H -0.30063000 1.84001200 0.86691500<br>H -5.74629600 -1.03663000 0.09062000<br>O -5.77936900 -0.15312900 -1.55721200<br>O -5.34183700 -1.17585200 -0.78477100	
**Name**	**TS-MO-O7-H-OOH (Gas phase)**
Cartesian Coordinates	Frequency and Energy
O 3.97925400 1.27588700 0.31394900<br>C 3.53747900 1.76148000 -0.97031700<br>C 2.10655000 2.22881300 -0.72034900<br>C 1.61638700 1.14517900 0.25248100<br>C 2.92272000 0.80649700 1.00752600<br>O 1.27004300 0.04706000 -0.59067700<br>C 1.11462300 -1.21620200 0.01592300<br>C 2.35618600 -2.09332800 -0.13297000<br>C -0.10876200 -1.90178400 -0.60168800<br>C -1.35376800 -1.13256100 -0.25383200<br>O 3.40741800 -1.41876100 -0.59658900<br>C 4.65000900 -2.13131500 -0.59014600<br>O 2.37359800 -3.25412200 0.17151900<br>O 3.02214600 0.22403800 2.04495300<br>O 2.06237500 3.52415000 -0.16847700<br>C -1.94456000 -1.30504300 1.01548900<br>C -3.03172600 -0.54650700 1.39420300<br>C -3.56910200 0.40728700 0.50862400<br>C -2.97586600 0.56928100 -0.77236700<br>C -1.87116900 -0.19556900 -1.13687600	Zero-point correction= 0.339858 (Hartree/Particle)<br>Thermal correction to Energy= 0.365031<br>Thermal correction to Enthalpy= 0.365975<br>Thermal correction to Gibbs Free Energy= 0.281448<br>Sum of electronic and zero-point Energies= -1334.666368<br>Sum of electronic and thermal Energies= -1334.641195<br>Sum of electronic and thermal Enthalpies= -1334.640251<br>Sum of electronic and thermal Free Energies= -1334.724778

O -3.47913800 1.47283000 -1.61679100<br>O -4.60943800 1.16683800 0.80586800<br>C 0.48556000 1.54395400 1.17517600<br>H 3.57452300 0.93628200 -1.68245200<br>H 4.20046000 2.57134800 -1.26782900<br>H 1.49511700 2.23982900 -1.62122500<br>H 0.96410400 -1.12013000 1.09751700<br>H 0.02904800 -1.94663000 -1.68452500<br>H -0.15408700 -2.92259000 -0.21761400<br>H 5.39293900 -1.42625300 -0.95289200<br>H 4.88618500 -2.44854700 0.42531100<br>H 4.58638100 -3.00311600 -1.24068000<br>H 2.72954300 3.60579900 0.52143300<br>H -1.52863200 -2.03825900 1.69866000<br>H -3.49451200 -0.65460400 2.36858100<br>H -1.42397800 -0.03933300 -2.11125600<br>H -4.22247500 1.90767300 -1.17191600<br>H -5.53122900 0.57320300 0.63563300<br>H 0.26717600 0.74704600 1.88783400<br>H 0.75041000 2.43771300 1.73977000<br>H -0.40717700 1.75215400 0.58197700<br>H -5.18323900 -1.79516200 -0.04976400<br>O -6.32181700 -0.34564400 0.22196200<br>O -5.52168200 -1.06946500 -0.59844300	
**Name**	**TS-MO-O7-H-OOH (Pentyl ethanoate)**
Cartesian Coordinates	Frequency and Energy
O 4.06039600 1.16872000 0.25808800<br>C 3.58696500 1.72995400 -0.98872800<br>C 2.17979000 2.22575300 -0.67371800<br>C 1.69521300 1.14480100 0.30765000<br>C 3.02179200 0.73394600 0.98680500<br>O 1.25429200 0.07868900 -0.53144900<br>C 1.08148400 -1.18880800 0.06118300<br>C 2.31660200 -2.07596900 -0.07763700<br>C -0.13341900 -1.85600000 -0.59465000<br>C -1.38013300 -1.08106200 -0.26455000<br>O 3.30730600 -1.48187100 -0.73361200<br>C 4.54982500 -2.19765000 -0.77767500<br>O 2.37026000 -3.18807700 0.37867300<br>O 3.15127700 0.12154100 2.00831700<br>O 2.17824800 3.52124100 -0.11716600<br>C -1.97403900 -1.23420600 1.00634900<br>C -3.07086800 -0.48118300 1.36536400<br>C -3.61452900 0.44688100 0.45696100<br>C -3.01721000 0.59245200 -0.82278200<br>C -1.90294700 -0.16646300 -1.16859800<br>O -3.52720100 1.47421900 -1.69011500<br>O -4.66264500 1.20926000 0.73937700<br>C 0.63169300 1.57615000 1.29252000<br>H 3.58033700 0.93918900 -1.73935600<br>H 4.26600200 2.53106100 -1.27364400<br>H 1.53894800 2.25509600 -1.55370500<br>H 0.90287600 -1.10708800 1.13864000<br>H 0.02680700 -1.89270800 -1.67480300	Zero-point correction= 0.339358 (Hartree/Particle)<br>Thermal correction to Energy= 0.364480<br>Thermal correction to Enthalpy= 0.365425<br>Thermal correction to Gibbs Free Energy= 0.281612<br>Sum of electronic and zero-point Energies= -1334.701276<br>Sum of electronic and thermal Energies= -1334.676154<br>Sum of electronic and thermal Enthalpies= -1334.675210<br>Sum of electronic and thermal Free Energies= -1334.759023

H	-0.20530600	-2.87827700	-0.21786200	
H	5.23525300	-1.56426600	-1.33525700	
H	4.92052500	-2.35924200	0.23510400	
H	4.41750700	-3.15464400	-1.28232900	
H	2.84316700	3.58060900	0.57970600	
H	-1.55365300	-1.94975500	1.70541700	
H	-3.53560000	-0.58075200	2.34030200	
H	-1.45379100	-0.02955900	-2.14554900	
H	-4.27374000	1.91928700	-1.25936000	
H	-5.56539800	0.60577100	0.62446800	
H	0.37630700	0.76153300	1.97238500	
H	0.97947400	2.41626300	1.89491500	
H	-0.26319800	1.88129200	0.74491200	
H	-5.22011500	-1.79544900	0.08709800	
O	-6.38757400	-0.34857900	0.26432000	
O	-5.59907500	-1.12888600	-0.51294100	
**Name**				**TS-MO-O7-H-OOH (Water)**
Cartesian Coordinates				Frequency and Energy
O	4.02216900	1.24759800	0.18999100	Zero-point correction= 0.339493 (Hartree/Particle)
C	3.51102400	1.84547700	-1.02985800	Thermal correction to Energy= 0.364455
C	2.07131300	2.23638300	-0.70879500	Thermal correction to Enthalpy= 0.365399
C	1.66176100	1.14809800	0.30082400	Thermal correction to Gibbs Free Energy= 0.282065
C	3.01785300	0.78213500	0.93660600	Sum of electronic and zero-point Energies= -1334.712414
O	1.22995300	0.05419700	-0.51270900	Sum of electronic and thermal Energies= -1334.687453
C	1.10385500	-1.21214000	0.10416000	Sum of electronic and thermal Enthalpies= -1334.686509
C	2.36384100	-2.05341600	-0.05479300	Sum of electronic and thermal Free Energies= -1334.769843
C	-0.10743600	-1.92047000	-0.51183400	
C	-1.35301400	-1.14166600	-0.18640200	
O	3.30206200	-1.45569900	-0.76883800	
C	4.57287500	-2.12479700	-0.84879400	
O	2.48118800	-3.14834000	0.44710300	
O	3.20146400	0.15999700	1.95176800	
O	1.96207500	3.54877600	-0.19750900	
C	-1.86259100	-1.17044000	1.12982600	
C	-2.95113500	-0.40313600	1.47749800	
C	-3.56346900	0.42436400	0.51880900	
C	-3.05703800	0.43906900	-0.80615000	
C	-1.95347800	-0.33900300	-1.14546900	
O	-3.64486900	1.20726200	-1.74262900	
O	-4.60297700	1.20913200	0.81282800	
C	0.62261800	1.55202200	1.32174900	
H	3.56708000	1.09535400	-1.81861400	
H	4.13752900	2.70244200	-1.26442000	
H	1.43109100	2.18760000	-1.58773200	
H	0.94853400	-1.12049700	1.18370000	
H	0.03512400	-1.99471900	-1.59185300	
H	-0.16278100	-2.92700500	-0.09216500	
H	5.20585800	-1.47426000	-1.44582500	
H	4.98368700	-2.24718900	0.15334000	
H	4.45391600	-3.09406000	-1.33137600	
H	2.57048700	3.66801400	0.54337800	
H	-1.38153200	-1.80124600	1.86972100	
H	-3.35665100	-0.41071100	2.48258400	
H	-1.57445200	-0.30291300	-2.16058100	

H -4.37705100 1.69760900 -1.33657900<br>H -5.50874600 0.69444900 0.52784200<br>H 0.42653900 0.73655000 2.02004600<br>H 0.97088300 2.41172600 1.89566700<br>H -0.30420900 1.81581100 0.80573000<br>H -5.32075400 -1.77706900 0.21900600<br>O -6.35506200 -0.22551600 0.04765300<br>O -5.49680500 -1.12675100 -0.48467300	
**Name**	**TS-MO-O7-RAD-O6-OOH (Pentyl ethanoate)**
Cartesian Coordinates	Frequency and Energy
O 3.75636600 1.17891400 -0.60773000<br>C 2.97678500 1.35397200 -1.81429500<br>C 1.65214300 1.93313600 -1.32956800<br>C 1.47243700 1.19124900 0.00554200<br>C 2.94159200 0.98359700 0.43870200<br>O 0.91488700 -0.07199800 -0.36040100<br>C 0.96671900 -1.10744500 0.59515600<br>C 2.18996900 -2.00661300 0.43044500<br>C -0.32118500 -1.93342700 0.46170600<br>C -1.50045800 -1.05584000 0.75594400<br>O 2.96492400 -1.63931800 -0.58260300<br>C 4.20943200 -2.34354000 -0.70920700<br>O 2.41076500 -2.93607700 1.16135400<br>O 3.33740700 0.69233500 1.53109100<br>O 1.70094000 3.33353100 -1.17884300<br>C -1.95649300 -0.87727500 2.05661200<br>C -2.97654000 0.02473300 2.33436300<br>C -3.63858600 0.78883600 1.30344800<br>C -3.14598900 0.58966200 -0.07254200<br>C -2.09763900 -0.30393800 -0.30071700<br>O -3.71869600 1.21498300 -1.05605100<br>O -4.56828800 1.56742000 1.56026200<br>C 0.63569100 1.90227000 1.04535800<br>H 2.84729400 0.37796100 -2.28269800<br>H 3.52251900 2.03019300 -2.46926600<br>H 0.82508300 1.71189200 -2.00289800<br>H 1.02698300 -0.71170200 1.61455200<br>H -0.38069000 -2.33314100 -0.55400300<br>H -0.27143400 -2.76381500 1.16770800<br>H 4.70784200 -1.90742400 -1.57119400<br>H 4.80876600 -2.19917400 0.19029100<br>H 4.02791600 -3.40625800 -0.86924000<br>H 2.50708000 3.58677000 -0.71255100<br>H -1.49617900 -1.43604600 2.86482300<br>H -3.31524600 0.18529500 3.35191700<br>H -1.70743800 -0.40371800 -1.30650700<br>H -4.06400200 0.43139400 -1.87096300<br>H 0.56860000 1.31405900 1.96233900<br>H 1.07274200 2.86820600 1.30075200<br>H -0.36875400 2.06714000 0.64739100<br>H -4.55041100 -1.99422400 -1.31362900<br>O -4.49632300 -0.51329800 -2.44559200<br>O -3.86530000 -1.57715900 -1.86464500	Zero-point correction= 0.325593 (Hartree/Particle)<br>Thermal correction to Energy= 0.350718<br>Thermal correction to Enthalpy= 0.351662<br>Thermal correction to Gibbs Free Energy= 0.266577<br>Sum of electronic and zero-point Energies= -1334.052264<br>Sum of electronic and thermal Energies= -1334.027139<br>Sum of electronic and thermal Enthalpies= -1334.026195<br>Sum of electronic and thermal Free Energies= -1334.111279
**Name**	**TS-MO-O6-RAD-O7-OOH (Pentyl ethanoate)**

Cartesian Coordinates	Frequency and Energy
O 4.00803900 1.14347300 0.43895500<br>C 3.58799000 1.76040200 -0.80096700<br>C 2.16473700 2.23422300 -0.52706800<br>C 1.64397800 1.10623900 0.38008700<br>C 2.94134100 0.66495200 1.09494000<br>O 1.24150000 0.07872700 -0.52629400<br>C 1.05791500 -1.21809900 -0.00436000<br>C 2.30771700 -2.08716100 -0.12486800<br>C -0.11550500 -1.86250400 -0.75355400<br>C -1.36781500 -1.07154300 -0.49124300<br>O 3.32012800 -1.45456200 -0.70589900<br>C 4.57315600 -2.15407100 -0.72295500<br>O 2.34960200 -3.21883400 0.28112200<br>O 3.02732700 -0.00233400 2.08609900<br>O 2.12886300 3.50121400 0.08944000<br>C -2.14841700 -1.32768300 0.62451200<br>C -3.26826800 -0.51615500 0.94301600<br>C -3.64325700 0.56023700 0.13698600<br>C -2.85348300 0.85106400 -1.07361100<br>C -1.70885800 0.00862100 -1.31652400<br>O -3.15104500 1.77719400 -1.84431200<br>O -4.68559800 1.28503900 0.40936400<br>C 0.53775700 1.49050600 1.33722900<br>H 3.62097600 1.00544000 -1.58703500<br>H 4.27403900 2.57682500 -1.01736000<br>H 1.56284800 2.30171900 -1.43234200<br>H 0.82047300 -1.19096100 1.06458000<br>H 0.11396300 -1.87696500 -1.82208200<br>H -0.22627700 -2.89043300 -0.40532500<br>H 5.27673200 -1.48588400 -1.21319000<br>H 4.89477400 -2.36194900 0.29803200<br>H 4.47859000 -3.08576900 -1.28063400<br>H 2.76429000 3.53261100 0.81509700<br>H -1.89214400 -2.14796200 1.28664600<br>H -3.83017500 -0.70988700 1.84829000<br>H -1.10360700 0.24886600 -2.18418900<br>H -5.63181000 0.59982200 0.54514300<br>H 0.24276300 0.64050300 1.95520800<br>H 0.86330700 2.28956600 2.00439000<br>H -0.32622800 1.84135200 0.76734400<br>H -5.86025000 -1.71845500 -0.49831200<br>O -6.48164800 -0.22700200 0.43108800<br>O -5.79419300 -1.40824000 0.42119200	Zero-point correction= 0.325437 (Hartree/Particle)<br>Thermal correction to Energy= 0.350612<br>Thermal correction to Enthalpy= 0.351556<br>Thermal correction to Gibbs Free Energy= 0.266205<br>Sum of electronic and zero-point Energies= -1334.050668<br>Sum of electronic and thermal Energies= -1334.025493<br>Sum of electronic and thermal Enthalpies= -1334.024549<br>Sum of electronic and thermal Free Energies= -1334.109900
**Name**	**TS-MO-O7-RAD-O6-OOH (Water)**
Cartesian Coordinates	Frequency and Energy
O 3.83739400 1.16144100 -0.43351300<br>C 3.08901100 1.66799600 -1.56878700<br>C 1.74752700 2.13423200 -1.00432300<br>C 1.56737100 1.22026400 0.22292000<br>C 3.02225200 0.86489500 0.57857300<br>O 0.92924100 0.05178300 -0.30219800<br>C 0.92067700 -1.11432400 0.49669700<br>C 2.15056300 -1.98649400 0.27528000	Zero-point correction= 0.325965 (Hartree/Particle)<br>Thermal correction to Energy= 0.350155<br>Thermal correction to Enthalpy= 0.351099<br>Thermal correction to Gibbs Free Energy= 0.268936<br>Sum of electronic and zero-point Energies= -1334.065255<br>Sum of electronic and thermal Energies= -1334.041066<br>Sum of electronic and thermal Enthalpies= -1334.040122<br>Sum of electronic and thermal Free Energies= -1334.122285

C	-0.35155200	-1.89338900	0.13480400	
C	-1.54367200	-1.06934600	0.51592300	
O	2.84656400	-1.63237400	-0.79127100	
C	4.09167400	-2.32255300	-1.00232300	
O	2.43996200	-2.90115700	1.01146700	
O	3.41627300	0.36670500	1.60262200	
O	1.73388000	3.51177000	-0.69516600	
C	-1.94236700	-0.96675800	1.84769200	
C	-2.98176700	-0.12945300	2.21409100	
C	-3.71532300	0.64842800	1.24602400	
C	-3.28468000	0.53813200	-0.15159300	
C	-2.22027500	-0.30279700	-0.47828200	
O	-3.94363100	1.18095700	-1.07902800	
O	-4.67233200	1.38132000	1.57273800	
C	0.80575600	1.81319900	1.38587700	
H	2.97936000	0.85593700	-2.28690400	
H	3.66286400	2.48658900	-1.99678700	
H	0.93433100	1.96081100	-1.70669700	
H	0.90404500	-0.87592700	1.56403900	
H	-0.34706700	-2.09577800	-0.93803800	
H	-0.34926500	-2.83903500	0.68084800	
H	4.53360300	-1.86097400	-1.88076400	
H	4.73424200	-2.18781200	-0.13218900	
H	3.90498900	-3.38154600	-1.17626000	
H	2.48033000	3.72557000	-0.12023800	
H	-1.41734500	-1.53953900	2.60439400	
H	-3.28148000	-0.03326800	3.25154800	
H	-1.87656900	-0.33499200	-1.50523200	
H	-4.37445300	0.37382700	-1.83990300	
H	0.78286200	1.11994100	2.22833000	
H	1.28103100	2.73562000	1.72143900	
H	-0.21709400	2.03132500	1.06924400	
H	-4.55748300	-2.03339000	-1.16928600	
O	-4.80401800	-0.60478100	-2.35381200	
O	-4.02184400	-1.60560800	-1.86221200	
